# Ex Vivo Assessment of Natural Teeth Wear against Zirconia and Novel Glass-Fiber-Reinforced Composite Crowns in Primary Teeth by a Three-Dimensional Assessment Method

**DOI:** 10.1155/2021/9670982

**Published:** 2021-12-20

**Authors:** Abhinav Talekar, Gayatri Chaudhari, Sreekanth Kumar Mallineni, Sneha Kothare, Amol Patil, Prasad Musale, Yusuf Chunawala, Shikha Choubey

**Affiliations:** ^1^Department of Pediatric and Preventive Dentistry, M.A. Rangoonwala College of Dental Science and Research Center, Pune, India; ^2^Department of Preventive Dental Science, College of Dentistry, Majmaah University, AlMajmaah 11952, Saudi Arabia; ^3^Private Practitioner at Mumbai, Mumbai, India; ^4^Department of Pediatric and Preventive Dentistry, SMBT Institute of Dental Sciences & Research, Nasik, India

## Abstract

**Objectives:**

The main purpose of the study was to assess the material wear, antagonistic natural primary teeth wear, and microhardness of zirconia (ZR), a recently launched novel glass-fiber-reinforced composite crown (GFRC). The research question was, are these aesthetic crowns resulting in antagonistic natural primary tooth wear and the crown material itself?

**Methods:**

Forty-five primary canines were divided into three groups (15 per group) and mounted against Zr (Group A), GFRC (Group B), and natural teeth as control (Group C) in the wear test machine. All samples were assessed for surface wear with pre- and post-3-dimensional scanning. In addition, microhardness was assessed for all three groups.

**Results:**

The mean microhardness value for the Zr disc was 1157 ± 7 HV; for the GFRC disc, it was 29.35 ± 2 HV; while with natural teeth, it was 105 ± 4 HV. There was a statistically significant difference in teeth wear in the prescan and postscan in the natural tooth (*p* < 0.05) group, highly significant difference (*p* < 0.001) in the ZR group, and no significant difference in the GFRC group.

**Conclusion:**

There is more significant wear loss of glass-fiber-reinforced composite discs as compared to zirconia. In addition, the wear of the antagonistic tooth with zirconia and natural teeth is more remarkable than with GFRC. There is a vast difference of microhardness between natural teeth and zirconia (almost 10 times higher) which suggests further scope of study. *Clinical Relevance*. Pediatric dentistry deals with the transition of dentition from primary to permanent through mixed dentition. Selection of restorative material needs to be done cautiously when we are dealing with primary teeth and young permanent teeth as antagonistic teeth. Wear of the crown material itself and opposing natural teeth are essential factors that should be considered in selecting crowns in clinical practice. The present study results can be extrapolated to clinical practice, and the practitioner can consider various factors in selecting full-coverage crowns for primary teeth. The vast difference in aesthetic crowns and natural teeth microhardness indicates a further need for research. Additionally, there is no literature published for the recently launched GFRCs.

## 1. Introduction

In primary dentition, full-coverage restoration is indicated for extensive or multisurface carious lesions. Preformed stainless steel crowns (SSCs) have been used for this purpose since 1950 and are still considered the gold standard [1, 2]. Despite their known advantages and proven success, SSCs do not meet the aesthetic demands of recent times. These aesthetic demands led to various innovations such as modifications of SSC such as preveneered, composite facing, shell crowns, composite crowns, polycarboxylate crowns, celluloid crowns, and recently, zirconia crowns (ZR) [3–6]. On this aesthetic bandwagon, the clinical success of strip crowns and zirconia crowns is documented comparatively to other options [4, 6, 7]. However, ZR crowns have their disadvantages. These crowns are thicker than SSCs, requiring substantial tooth preparation, which may cause pulp exposure, may require passive seating when cementing, and cannot be modified to conform to the tooth [3, 6].

To overcome these drawbacks of ZR crowns, uphold the properties of SSCs and meet aesthetic demands, a new type of glass-fiber-reinforced composite (GFRC) crown has been recently introduced. Less aggressive tooth reduction and reduced time requirements compared to ZR crowns, the ability to modify crowns, aesthetics similar to ZR crowns, and greater cost-effectiveness are some of the advantages of these crowns over ZR crowns as enlisted by the manufacturer [8]. However, there is no literature available in this regard. Furthermore, very little is known about its wear properties.

Human dentition is subjected to continuous stress when in occlusion. This stress leads to wear of the enamel and additionally leads to modification of the periodontium. The amount of wear caused depends on varying enamel strengths [9], enamel and dentin thickness [10], and the completely different biting forces of adults and infants [11]. In children, there is an increase in the mean maximum bite force in the molar region throughout growth and development, irrespective of gender [12]. The Vickers hardness number (HV) of primary enamel was 106 (HV) and that of permanent enamel was 126 (HV) [13]. Mortimer found primary teeth to be less mineralized than permanent teeth, and Nelson et al. reported primary tooth enamel was thinner than permanent tooth enamel [14]. These factors make the primary enamel more prone to wear than permanent enamel [12–15].

The occlusion and constant biting forces result in the wear of the opposing tooth and subject any restorative material (filling or crown) to wear [11, 16, 17]. Additionally, the durability of crowns is known to differ for different materials [18]. In particular, when we are dealing with primary teeth, the selection of crown material has to be careful as these teeth are more prone to wear [12–15]. Considering all these factors, the current study was aimed to assess the crown material wear and antagonistic enamel wear in primary teeth with regularly used zirconia crowns and the recently launched novel glass-fiber-reinforced composite crowns (Figaro Crowns, Woodbury, MN, US) in primary teeth. The research question was, are these crowns resulting in antagonistic natural primary tooth wear and the crown material itself? The null hypothesis is that there was no wear in either the material of aesthetic crowns or the antagonistic natural teeth.

## 2. Materials and Methods

### 2.1. Ethical Approval and Study Design

This project was approved by the Institutional Ethical Committee, M. A. Rangoonwala College of Dental Sciences and Research Centre, Pune, India, according to the ethical standards laid down in the 1964 Declaration of Helsinki and its later amendments. The current study is designed according to CRIS guidelines.

#### 2.1.1. Materials

**Figure 1:**
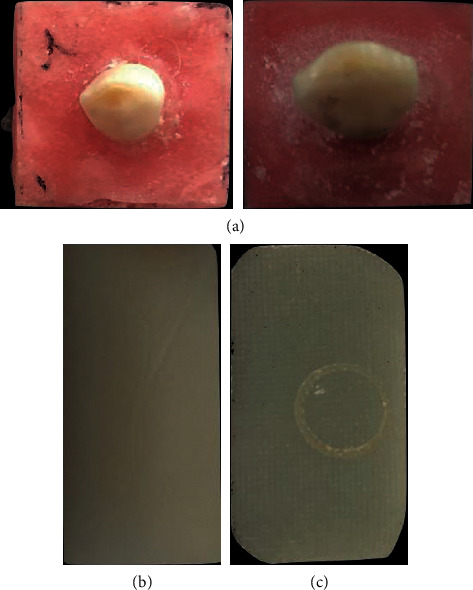
(a) Primary canine mounted, (b) zirconia crown material disc, and (c) glass-fiber-reinforced composite crowns crown material disc.

A cuboidal disc (20 mm × 10 mm × 5 mm) of GFRC (Figaro Crowns 2nd Generation Inc., Woodberry, USA) and the cuboidal disc of ZR (20 mm × 10 mm × 5 mm) (Kids-e-Dental, Mumbai, India) were supplied by manufacturers as per our recommendations. Prepared specimens were fixed with an acrylic resin (orthodontic resin, Dentsply, Philadelphia, PA, USA) using uniform moulds. A single operator prepared all specimens (Figure 1).

Forty-five primary canines lacking preexisting cuspal wear, caries, and fractures extracted for orthodontic reasons were included in the study out of 67 collected teeth after applying exclusion criteria. The extracted specimens were collected, stored in normal saline, sterilized, and handled according to the Occupational Safety and Health Administration (OSHA) guidelines and regulations. The primary canine specimens were prepared by mounting them in an acrylic resin (orthodontic resin, Dentsply, Philadelphia, PA, USA), measuring 20 mm × 20 mm × 10 mm, and approximately 5 mm cusps of the primary canine teeth were exposed (Figure 1(a)).

#### 2.1.2. Sample Size Calculation

The total sample size of 45 was derived from a preliminary power analysis of 80% per group, with a 5% and two-sided test level. These forty-five teeth are further divided (15 per group) into Group A (zirconia disc), Group B (GFRC disc), and Group C (natural teeth) (Figures 1(b) and 1(c)).

#### 2.1.3. Wear Test Machine

A wear test was conducted using a CS-4.8 masticator (two-body wear tester ID no: WT 21- PRAJ; R.P.M. of the machine, 350; India). In two chambers, a restorative material disc was placed on top and the antagonistic tooth was placed at the bottom using specimen holders. In the third chamber, the natural tooth was mounted on top and antagonistic natural teeth at the bottom as a control group. The masticatory force was established using previous studies that compared the wear of primary teeth against different dental materials [17, 19, 20]. To meet the greater masticatory forces, 5 kg were loaded, comparable to 50 N of chewing force [21, 22]. According to previous studies, 2,50,000 were conducted in overloading cycles [21–23]. Additionally, a thermodynamic condition similar to the natural oral environment was reproduced using a computer-controlled hot/cold water circulation system [20, 21].

#### 2.1.4. Microhardness Assessment

The microhardness of the two discs and natural teeth was ascertained using a microhardness tester (Microhardness Tester, Reichert Austria Make, Sr. No.363798, Reference Standard: ISO 6507) with a load of 50 g.

#### 2.1.5. Allocation and Blinding

The natural teeth specimens were collected after the wear taste machine and allocated into three groups as Group 1 against (Zr disc), Group 2 against (GFRC disc), and Group 3 against (natural tooth) making sure the data operator was not aware of it.

#### 2.1.6. Three-Dimensional Scanning

**Figure 2:**
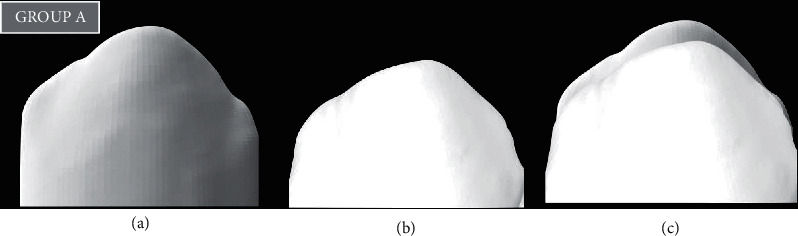
(a) 3D prescan of primary teeth with Group A, (b) 3D postscan of primary teeth with Group A, and (c) superimpose of prescan and postscan with Group A.

**Figure 3:**
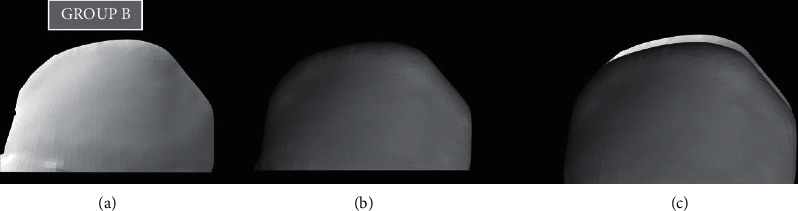
(a) 3D prescan of primary teeth with Group B, (b) 3D postscan of primary teeth with Group B, and (c) superimpose of prescan and postscan with Group B.

**Figure 4:**
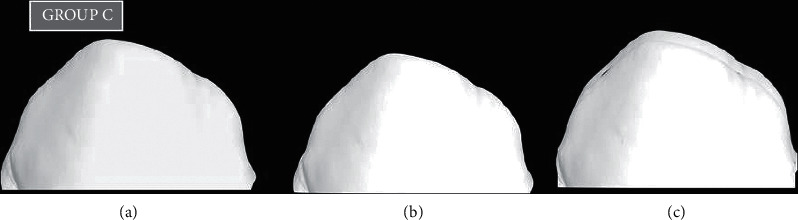
(a) 3D prescan of primary teeth with Group C, (b) 3D postscan of primary teeth with Group C, and (c) superimpose of prescan and postscan with Group C.

To measure the amount of tooth loss and material loss, all teeth and discs were scanned using a 3D scanner before and after testing [23, 24]. The 3D data obtained before and after testing were overlapped using 3D software (Dentacian Software, EZplant, Seoul, Korea). Worn areas were then separated using Boolean operations, and wear volumes were measured using 3D software (Dentacian Software, EZplant, Seoul, Korea) (Figures 2–4).

### 2.2. Statistical Analysis

The mean and standard deviation of the test parameters were calculated using SPSS (Ver. 17.0, SPSS, Chicago, IL, USA). The statistical significance of the mean difference of each parameter was tested with a significant level of 5% using one-way ANOVA and the Tukey test.

## 3. Results

The microhardness values of the ZR disc, GFRC disc, and natural teeth were obtained. The mean microhardness value for the Zr disc was 1157 ± 7 HV, for the GFRC disc, it was 29.35 ± 2 HV, while with natural teeth, it was 105 ± 4 HV (Table 1). Volume loss of all three groups was also demonstrated in (Table 1). The prewear test value for the ZR group was 7.53 (S.D.), and the postwear test value was 5.27 with a mean volume loss of around 2.26, which was statistically highly significant. (*p* < 0.001) (Figures 2(a)–2(c)). The prewear test value for the GFRC group was 7.55 (S.D), and the postwear test value was 6.69 with a volume loss of 0.86 with no significant difference (*p*=0.001) (Figure 3(a)–3(c)). While in the control group, the mean volume loss was 1.53, where the prescan and postscan values were 7.90 and 6.37, respectively, with a significant difference. (*p* < 0.05) (Figures 4(a)–4(c)). By applying the paired “t” test, the mean volume loss was significantly more after the wear test than the initial volume with the Zr group and the natural teeth group (Table 2).

## 4. Discussion

Wear can be defined as “the ultimate consequence of the interaction between surfaces, manifested in the gradual removal of material.” It is a natural process that occurs when surfaces move in contact [10]. Dental wear is defined as tooth loss or surface damage caused by direct contact between teeth or other materials. It occurs as a complex form of chemical and mechanical wear [25]. Although tooth wear and material wear are separate fields of research, the same fundamental processes are active in all types of structure. The study of dental materials must consider the wear resistance of the materials and their likely effect on the opposing teeth [10]. Thus, the present in-vitro study compared the amount of wear of ZR and GFRC material itself and the crowns' opposing tooth wear and microhardness.

Dental materials commonly undergo abrasive wear proportional to the hardness of the materials in contact, the geometry of the abrasive particles, the load, and the sliding distance [6, 25]. Restorative dental materials have different wear properties than natural teeth, so the durability of the restorative material changes [13, 18]. There exists a statistically significant difference (*p* < 0.05) in the percent wear of the two discs. The occlusal wear of GFRC is fifteen times that of ZR. Thus, the hypothesis that the material wear of ZR and GFRC is comparable is not accepted. Since both the discs were dimensionally identical and both groups were subjected to similar simulated forces in the experimental environment, the difference in the wear of the two materials can only be attributed to the hardness of the material [10].

The microhardness of GFRC (29.35 ± 2 HV) was much lower than primary enamel (105±4 HV), while that of the ZR crown (1157 ± 7 HV) was ten times higher than primary enamel. The findings are in agreement with prior studies reported in the literature [13, 26]. Kodaka and colleagues [27] stated that minor differences in the organic and inorganic content do not affect the overall microhardness, but significant mineral loss (as in extensive caries) results in gross changes in microhardness. Thus, clinically, the restorative materials must have more excellent wear resistance. However, since the microhardness of GFRC was lower than that of ZR, we conclude that a GFRC will be less wear-resistant than a ZR crown as a full-coverage restoration in a multisurface restoration.

The hardness of the material is also a reliable factor to determine the extent of antagonistic wear [26]. The critical hardness value of 45 HV is necessary for a composite resin to result in antagonistic enamel wear [28]. The microhardness of GFRC is less than that, and thus, the tooth volume loss of the antagonistic teeth in the GFRC group was much lower than that in the ZR group, and the difference between the two groups was statistically significant (Table 1). Thus, the hypothesis regarding antagonistic enamel wear is accepted. In this study, the total volumetric antagonistic enamel loss in the ZR group (2.26 ± 0.25) is comparable to Jung et al. [17]. It contrasts with the findings of Sripetchdanond and Leevailoj, who found no significant difference in the antagonistic enamel wear in zirconia and composite resin. They further claimed that the antagonistic enamel wear in composite resin is due to the protruding filler in the resin matrix. The higher antagonistic enamel loss in the ZR group was attributed to fatigue wear resulting in microscopic reticular cracks, a large number of small chipping flakes of enamel, and the formation of pit-like structures [29]. However, the depth of wear with the ZR crown was found to be clinically acceptable [30]. The control group before and after readings indicate a significant loss of tooth material, which indicates the probable reason is point contact of teeth.

In particular, the clinical implications of these findings, particularly excessive wear on the opposing tooth, are an abnormal load resulting in periodontal diseases, temporomandibular disorders due to the loss of vertical dimension, loss of centric occlusion, crossbite, and change of functional path during chewing, or masticatory muscle fatigue [23, 31, 32]. Although there are no reported cases of such discrepancies in the literature. Thus, wear of the crown material itself and opposing natural teeth are essential factors that should be considered in selecting crowns in clinical practice. Seghi suggested that a restorative dental material should have a wear degree similar to that of the enamel [33].

There are certain limitations to the present study. Firstly, only primary canines were considered for the study of natural tooth wear. Warren and colleagues reported disproportionate tooth wear of natural teeth in the primary dentition with the incisors and canines showing more significant wear than molars. Also, maxillary wear is more significant than mandibular wear. Morphologic and functional malocclusions also affect the degree of wear [34]. Furthermore, the enamel surfaces of the natural teeth were not evaluated for microcracks before testing. The presence or lack of enamel cracks would be an essential determinant for the extent of wear since enamel wear occurs due to the formation and propagation of surface and subsurface enamel cracks eventually resulting in structural loss. Lanza stated that there is a need for a standardized tribological test with proper wear measurement protocol in dental tribology. Comparison of tribological properties with different or same restorative materials under different operative conditions and predicting outcomes with clinical performances of the same is challenging [35].

The other limitation of this study is the use of two wear test machines. Wassell stated the disadvantage of lack of standardization in this method and suggested steatite as an alternative and close substitute to enamel [36].

The means of maximum occlusal bite force (MOBF) for the different dentition stages were 176 N in the early primary stage, 240 N in the late primary stage, 289 N in the early mixed stage, 433 N in the late mixed stage, and 527 N in the permanent dentition stage, respectively. The MOBF increased with age, and gender differences were detected in the late primary stage and both stages of mixed dentition. Height and age were significantly correlated with the MOBF in all dentition stage groups except the early primary stage [37]. These changes occurring in the dentition were not considered in the present study. A constant load of 50N was considered during the simulation in the present study.

The literature about primary tooth wear against aesthetic crowns is scarce. The current study focuses on the same part with the inclusion of well-documented and aesthetic crowns in one group, recently launched novel GFRCs on the other hand, about which there are not many documented studies, and natural primary teeth in the control group. The other novelties of this particular paper are the use of a three-dimensional scanning method which is a more precise way as compared to other conventional methods. A very recently published clinical trial by Talekar et al. stated that the GFRC had occlusal wear with 18-month follow-up [38]. The results of the same trial are correlated with the current paper.

Tooth wear is a cumulative multifaceted process, with an interplay of mechanical forces and chemical dissolution [39]. During the masticatory process, the natural teeth and crowns are subjected to various erosive, abrasive, and attritive wear in an acidic environment. There is conflicting evidence to suggest that the diet, especially an acidic diet, impacts the wear pattern of the primary dentition [10]. The in vitro simulated oral environment cannot consider these dynamic changes. Furthermore, representative rectangular discs of the restorative materials were used instead of the actual crowns in the study, and thus, the surface characteristics of the crowns in the oral environment cannot be recreated in the present study. The other factor that needs to be considered is the use of natural lubricant as an alternative to water and the profilometric investigation method, as these methods help to determine the difference in wear of restorative material as well as opposing natural teeth in a more precise way [40, 41]. In light of these shortcomings, the present study results can be extrapolated to clinical practice, and the practitioner can consider various factors in selecting full-coverage crowns for primary teeth. Studies with in vitro comparisons of commercially available crowns and long-term randomized control trials are necessary in this regard.

## 5. Conclusion

Based on the results of this study, despite its limitations, the following conclusions can be made:

1. There is more significant wear loss of glass-fiber-reinforced composite discs as compared to zirconia.
2. The wear of the antagonistic tooth with zirconia and natural teeth is more significant than with GFRC.
3. The microhardness value of zirconia is ten times higher than natural teeth. This difference indicates the further scope of research in pediatric dentistry tribology with a more precise standardized method.
4. The present study results can be extrapolated to clinical practice, and the practitioner can consider various factors in selecting full-coverage crowns for primary teeth.

## Figures and Tables

**Table 1:** Microhardness of glass-fiber-reinforced composite crowns, zirconia discs, and natural teeth.

Sample type	Microhardness in HV		
	Reading 1	Reading 2	Mean
Zirconia crown	1150	1164	1157
Glass-fiber-reinforced composite crowns	29.15	29.56	29.35
Natural teeth	101	109	105

**Table 2:** Volume loss of the antagonistic teeth against glass-fiber-reinforced composite crowns, zirconia crowns, and natural teeth groups.

Sample type	Prewear mean (SD)	Postwear mean (SD)	Volume loss	Paired “t” test	*p*value significance
Zirconia crowns	7.53	5.27	2.26	33.362	*p* < 0.001^∗∗^
Glass-fiber-reinforced composite crowns	7.55	6.69	0.86	13.18	*p*=0.001
Natural teeth	7.90	6.37	1.53	22.782	*p* < 0.05^*∗*^

^∗∗^ *p* < 0.001 value indicates statistically highly significant difference; ^*∗*^*p* < 0.05 value indicates statistically significant difference.

## Data Availability

Data supporting this research article are available from the corresponding author on reasonable request.
